# Impacts of an Interdisciplinary Developmental Follow-Up Program on Neurodevelopment in Congenital Heart Disease: The CINC Study

**DOI:** 10.3389/fped.2020.539451

**Published:** 2020-10-06

**Authors:** Solène Fourdain, Laura Caron-Desrochers, Marie-Noëlle Simard, Sarah Provost, Amélie Doussau, Karine Gagnon, Lynn Dagenais, Émilie Presutto, Joëlle Prud'homme, Annabelle Boudreault-Trudeau, Ioana Medeleine Constantin, Béatrice Desnous, Nancy Poirier, Anne Gallagher

**Affiliations:** ^1^Sainte-Justine University Hospital Research Center, Montreal, QC, Canada; ^2^Department of Psychology, Université de Montréal, Montreal, QC, Canada; ^3^School of Rehabilitation, Université de Montréal, Montreal, QC, Canada; ^4^Clinique d'Investigation Neurocardiaque (CINC), Sainte-Justine University Hospital Center, Montreal, QC, Canada; ^5^Division of Neurology, Department of Pediatrics, La Timone Hospital, Marseille, France; ^6^Faculty of Medicine, Université de Montréal, Montreal, QC, Canada

**Keywords:** congenital heart disease, neurodevelopment, neurocardiac program, early intervention, interdisciplinary, preschool age, developmental assessment

## Abstract

**Objectives:** This study investigates the impact of an early systematic interdisciplinary developmental follow-up and individualized intervention program on the neurodevelopment of children with complex congenital heart disease (CHD) who required cardiac surgery.

**Study Design:** We prospectively enrolled 80 children with CHD: 41 were already followed at our neurocardiac developmental follow-up clinic from the age of 4 months, while 39 were born before the establishment of the program and therefore received standard health care. We conducted cognitive, motor, and behavioral assessments at 3 years of age. We used one-way multivariate analyses of variance to compare the neurodevelopmental outcome of both groups.

**Results:** Between-group analyses revealed a distinct neurodevelopmental profile with clinically significant effect size (*P* < 0.001, partial η^2^ = 0.366). Children followed at our clinic demonstrated better receptive language performances (*P* = 0.048) and tended to show higher scores on visuo-constructive tasks (*P* = 0.080). Children who received standard health care exhibited greater performances in working memory tasks (*P* = 0.032). We found no group differences on global intellectual functioning, gross and fine motor skills, and behaviors. Referral rates for specific remedial services were higher in patients followed at our neurocardiac clinic compared to the historical cohort (*P* < 0.005).

**Conclusions:** Overall, the impact of the developmental follow-up and individualized intervention program on neurodevelopmental outcomes remains subtle. Nevertheless, results, although limited by several factors, point toward an advantage for the children who took part in the program regarding receptive language skills over children who received standard health care. We hypothesize that group differences may be greater with growing age. Further research involving larger cohorts is needed to clearly assess the effectiveness of neurocardiac developmental follow-up programs at school age.

## Introduction

Congenital heart disease (CHD) is the most common congenital anomaly, affecting up to 1% of live births (1–4), with the more severe cardiac malformations requiring surgery or catheter-based interventions to ensure survival (5, 6). Advances in prenatal diagnosis, surgical techniques, and medical therapies have led to a significant rise in the survival rates of children with CHD (5, 7). This, however, has been associated with an increase in long-term neurodevelopmental comorbidity (7), with up to 50% of children with CHD presenting impairment in motor, language, and/or cognitive functions (8–19), along with behavioral and psychosocial maladjustment (20). These comorbidities generally limit academic achievement, employability, earnings, and insurability, and ultimately reduce the quality of life of patients and their families (10, 20–23).

**Figure 1:**
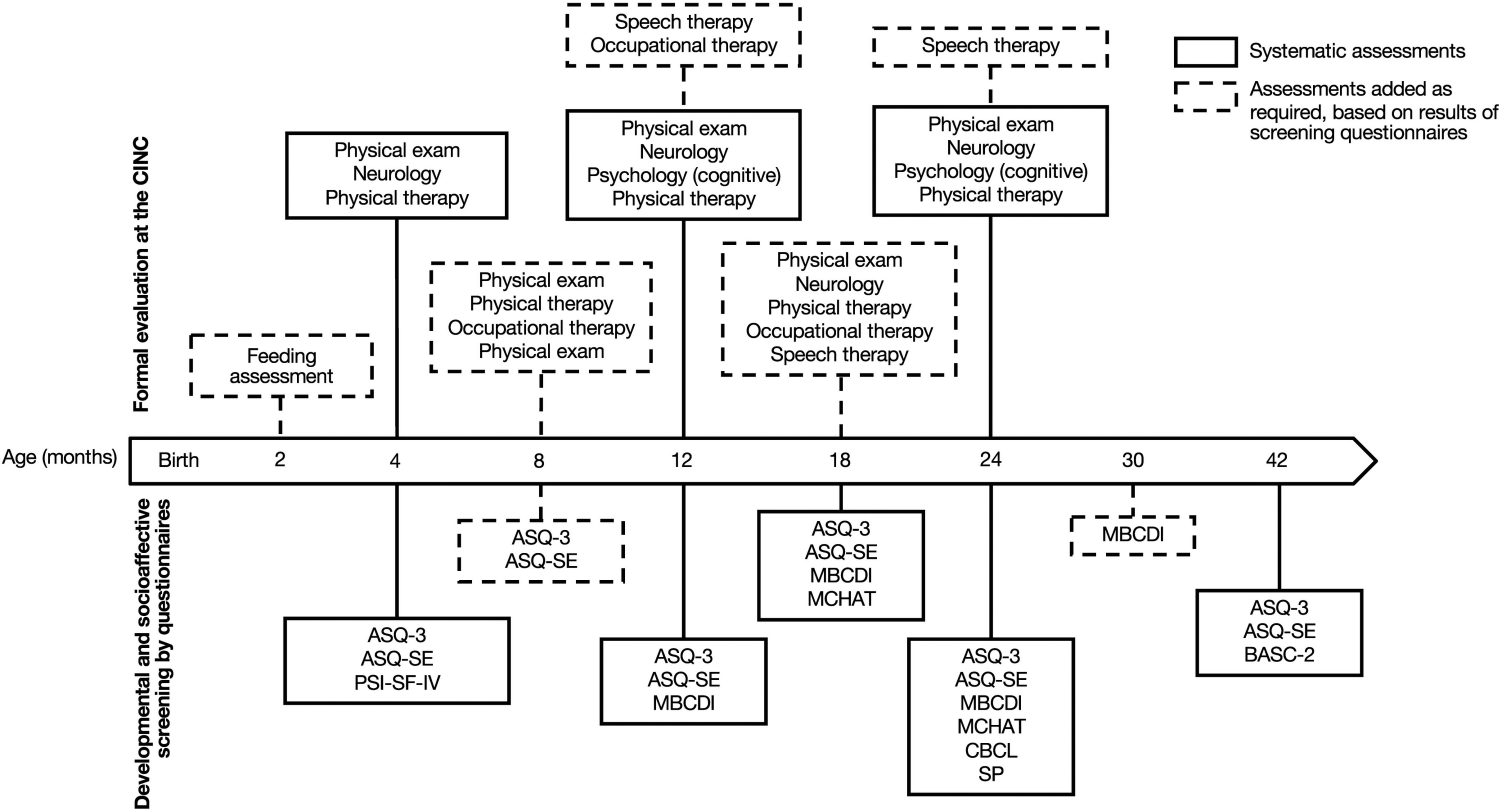
Timeline of developmental follow-up from 4 to 42 months of age *Clinique d'Investigation Neurocardiaque* (CINC). Systematic screening and assessments are represented in plain lines; additional examinations based on screening are represented in dotted lines. ASQ-3, Ages and Stages Questionnaire, Third edition; ASQ-SE, Ages and Stages Questionnaire: Social–emotional; MBCDI, MacArthur–Bates Communicative Development Inventories; MCHAT, Modified Checklist for Autism in Toddlers; BASC-2, Behavioral Assessment System for Children, Second Edition; SP, Sensory profile; CBCL, Child Behavior Checklist.

Given the aforementioned comorbidities, several studies have highlighted the need for an early and close developmental follow-up of children with CHD (24–29). In 2012, the American Heart Association and the American Academy of Pediatrics recommended systematic developmental surveillance, screening, and evaluation of patients with CHD throughout childhood to promote early diagnosis, implement relevant supportive strategies, and ultimately improve neurodevelopmental prognosis (30). Subsequently, many cardiac developmental follow-up programs have emerged in pediatric centers worldwide (26, 30, 31). At the Sainte-Justine University Hospital Center (Montreal, QC, Canada), we founded the *Clinique d'Investigation Neurocardiaque* (CINC) in 2013 to provide early systematic and interdisciplinary developmental follow-up for all children with critical CHD without genetic syndrome (e.g., trisomy 21). The establishment of the CINC program (i.e., identification of systematic time points for assessments, selection of assessment tools, recruitment of clinicians, etc.) is grounded on evidence from literature on the CHD population and similar clinical populations (e.g., preterm), and is gradually refined and adjusted according to probative research findings and hospital resources (19). The CINC interdisciplinary team is composed of a nurse practitioner coordinator, a pediatric cardiac surgeon, pediatric neurologists, cardiologists, developmental pediatricians, physical therapists, occupational therapists, a psychologist, a speech-language pathologist, and a nutritionist. Our program begins at the age of 4 months and consists of systematic and standardized developmental assessments at multiple pre-established ages. These include neurological and physical exams, motor and cognitive assessments, as well as socio-affective and behavioral screenings using parental questionnaires. While direct participation of professionals varies across assessments (e.g., 4-month neurologic exams are performed by developmental pediatricians or pediatric neurologists and 24-month cognitive and motor assessments are conducted by a neuropsychologist), all clinicians are invited to interdisciplinary clinical meetings, thus having a potential indirect involvement with every patient regardless of age. The timeline of the CINC developmental follow-up program is presented in Figure 1.

Early generalized and systematic intervention is also offered. It includes educational support to the child's family, such as information about infant growth and development, explanations of child behavior, and feedback from a professional on the parents' interaction with the child. Children may also obtain additional care according to their specific needs, therefore based on the results of the systematic assessments. This additional care consists of individualized recommendations to parents for educational activities, daily home exercises, or direct intervention with a therapist. Data from the scientific literature and the clinical experience of the CINC professionals have led to establish fixed criteria to determine the child's eligibility for further intervention (19, 32, 33). For instance, a child performing below the 10th percentile on the Alberta Infant Motor Scale (34) at 4 months will be considered at risk of gross motor delay (32, 33) and will receive individual sessions for motor intervention with the physical therapist (35). Similarly, a child with a score below the 2nd percentile in one scale of the MacArthur–Bates Communicative Development Inventories (MBCDI) (36), at the 10th percentile on the Communicative Gestures scale of the MBCDI, or below the 10th percentile in all of its scales will receive an extensive language assessment by a speech-language pathologist to determine the needs for speech therapy (19). A score in the monitoring zone or below the cutoff at the Ages and Stages Questionnaire, Third Edition (37), will also lead to a referral to a speech-language pathologist for evaluation. Finally, a child showing a failure to thrive, that is, an insufficient weight gain for age, an inappropriate weight loss, or a weight inferior to the 2nd percentile according to standards of growth, will be referred to a nutritionist.

In 2017, we published a case study (38) showing significant motor improvement in a 12-month-old girl with complex CHD who received early intervention with a physical therapist as part of the CINC program. Recently, Fourdain and colleagues described a substantial improvement of gross motor abilities in CINC patients aged up to 24 months, particularly in children at risk of motor delay who received physical therapy on a regular basis (35). Although these results suggest a positive impact of the CINC program, they could also be associated with spontaneous recovery after surgery (39, 40). Further research is therefore needed to quantify the benefits of neurocardiac follow-up programs on neurodevelopment. To this end, this study aimed to assess the impact of the CINC early systematic interdisciplinary developmental follow-up and individualized intervention program compared to standard health care on motor, cognitive, and behavioral development in 3-year-old children with critical CHD.

## Materials and Methods

### Patient Population

Since 2013, families of Sainte-Justine University Hospital Center's patients presenting with moderate to severe CHD requiring cardiac surgery have been offered a referral to the interdisciplinary neurocardiac clinic [*Clinique d'Investigation Neurocardiaque* (CINC)]. Children with genetic syndromes (e.g., trisomy 21) and children with severe or profound intellectual disability were not referred to the CINC as they already received specialized services in thematic clinics or rehabilitation centers. Since its opening, 16 (4%) families of the patients referred to the CINC declined the developmental interdisciplinary follow-up. Reasons for declining included being followed in another pediatric hospital in the Montreal area for 3 (19%), being followed in another specialized clinic in the Sainte-Justine University Hospital Center for 2 (13%), having been referred early to a pediatric rehabilitation center for 1 (6%), or being satisfied with the standard health care for 1 (6%). Nine (56%) families did not specify any reason for refusing the CINC follow-up. Currently, we provide early systematic and interdisciplinary developmental follow-ups to 338 children with CHD.

For this research study, we prospectively recruited a cohort of 43 children with complex CHD, followed longitudinally at the CINC. A total of 59 CINC patients were approached to participate in the present study: 14 (23.7%) did not respond to the invitation, 2 (3.4%) refused to participate due to lack of time, and 43 (72.9%) accepted the invitation and were scheduled for a research interdisciplinary standardized assessment at 3 years of age. Two (4.7%) children did not offer sufficient collaboration during the assessment to gather valid data. This resulted in 41 children whose data were included in this study and constituted the *Surveillance Group*. Among these, 5 (12.2%) did not attend the 4-month-old evaluation, 2 (4.9%) did not attend the 12-month assessment, and 2 (4.9%) did not attend the 24-month follow-up, with all children being present for at least two of these assessments.

To compare neurodevelopmental outcomes at the age of 3, we prospectively recruited a group of children with moderate to severe CHD who were born before the CINC was established and, as such, were not followed within the clinic's framework. These children received standard health care (pre-established follow-ups with the cardiologist and regular medical appointments with the pediatrician or family physician). As for the CINC referral criteria, we only included children who did not present genetic syndrome or severe or profound intellectual disability. Among the 63 eligible families contacted to participate in the study, 4 (6.3%) did not respond to the invitation, 19 (30.2%) refused to participate, and 40 (63.5%) accepted the invitation and were scheduled for the same research interdisciplinary standardized assessment at 3 years of age. The reasons provided when refusing to participate were lack of time for 6 (31.6%), long distance between home and the hospital for 6 (31.6%), stating that the child has no developmental delay for 4 (21%), parent or child having to undergo surgery for 2 (10.5%), and child being too afraid of hospitals for 1 (5.3%). One child did not offer sufficient collaboration during the assessment to gather valid data, resulting in 39 enrolled children who constituted the *Historical Control Group*.

**Figure 2:**
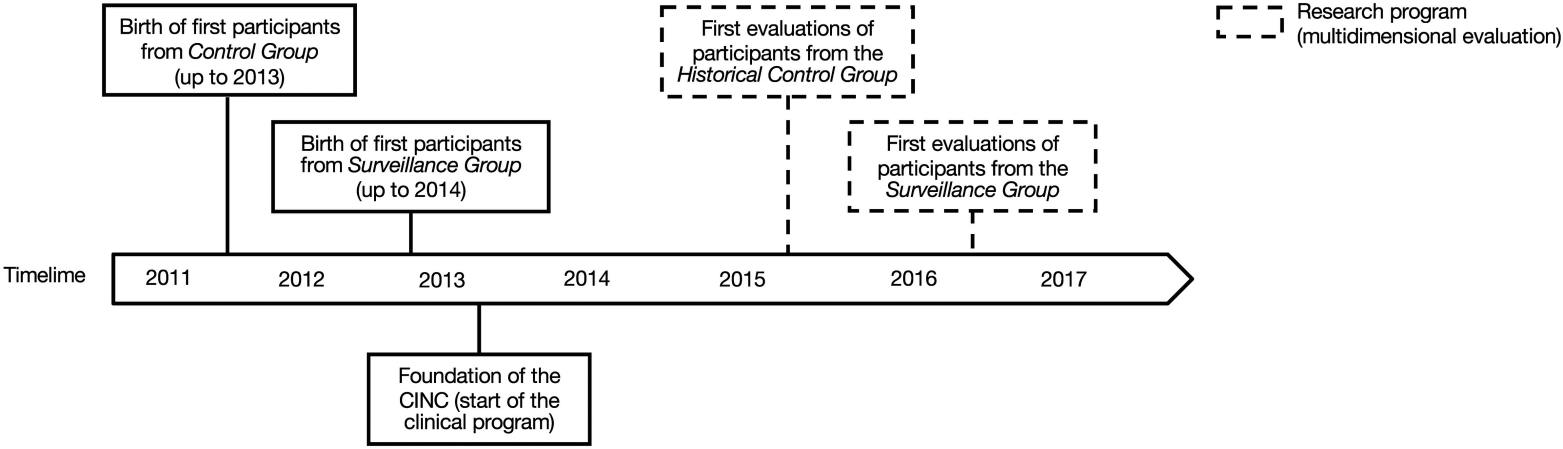
Timeline of participants' births (plain lines) and research assessments (dotted lines) for the *Historical Control Group* and the *Surveillance Group*.

A description of the timeline of participants' recruitment and assessment is presented in Figure 2. The study was approved by the institutional research ethics board of the Sainte-Justine University Hospital Center. All participants' parents gave written informed consent.

### Research Interdisciplinary Evaluations

Participants of the *Surveillance Group* and the *Historical Control Group* received an interdisciplinary neurodevelopmental evaluation as part of this research project. It included a cognitive assessment by neuropsychologists (SF and LCD), a gross motor assessment by a physical therapist (LD), a fine motor assessment by occupational therapists (KG and JP), and a behavioral assessment using a parental questionnaire. Interdisciplinary research assessments were conducted at the Sainte-Justine University Hospital Center during one full morning (90 min for the cognitive assessment, and 15 and 60 min for the fine and gross motor evaluations, respectively). Due to the young age of the participants, parents were present in the room with the child for the whole duration of the assessment. At the end of the meeting, professionals provided to parents a retroaction on the child's cognitive and motor performances, and behavioral skills during an interdisciplinary feedback session. Finally, the assessment findings and the resulting relevant recommendations were summarized in an interdisciplinary written report.

Cognitive assessment was performed using the Wechsler Preschool and Primary Scale of Intelligence, Fourth Edition (WPPSI-IV) (41). For 3-year-olds, it includes three verbal subtests (Receptive Vocabulary, Information, and Picture Naming), two visuospatial subtests (Block Design and Object Assembly), and two working memory subtests (Picture Memory and Zoo Locations). It provides a global cognitive score (Full-Scale IQ) as well as three primary index scales (Verbal Comprehension, Visual Spatial, and Working Memory). Mean performances of 10 [standard deviation (SD) = 3] and 100 (SD = 15) are expected on the subtest and scale levels, respectively.

Gross motor skills were assessed using the Peabody Developmental Motor Scale, Second Edition (PDMS-2) (42), which includes three gross motor subtests (Stationary, Locomotion, and Object Manipulation). It produces standard scores (Mean = 10; SD = 3) for each subtest as well as a Gross Motor Quotient (Mean = 100; SD = 15). Fine motor assessment was done using the Manual Dexterity scale of the Movement ABC, Second Edition (M-ABC-2) (43). It measures manipulative skills through three subtests (Posting Coins, Threading Beads, and Drawing Trail), each providing a standard score (Mean = 10; SD = 3). The M-ABC-2 also provides a Manual Dexterity composite score (Mean = 100; SD = 15).

Behavioral assessment was performed using the Parent Rating Scale of the Behavior Assessment System for Children, Second Edition (BASC-2), preschool version (44). It provides a *T* score (Mean = 50; SD = 10) for 12 syndrome scales and four composite scales (Externalizing Problems, Internalizing Problems, Behavioral Symptoms Index, and Adaptative Skills).

Perinatal, surgical, critical, and demographic variables were collected from medical records for participants of both groups. Anatomic CHD classification (45) and RACHS-1 scores (46) were extracted from the descriptions of heart defects and surgical procedures by a pediatric neurologist (BD). Information regarding the use of remedial services was collected through a parental report for both groups. For the *Surveillance Group*, additional information on remedial services was also retrieved from the CINC records.

### Statistical Analyses

Descriptive statistics [means, medians, SDs, and 95% confidence intervals (95% CI)] were calculated for continuous variables, and number of participants and percentages were calculated for dichotomous and categorical variables. Unpaired *t*-tests, Mann–Whitney *U*-tests, and Chi-squared tests were used to compare both groups on continuous, categorical, and dichotomous variables, respectively.

One-way multivariate analyses of variance (MANOVA) were computed for intergroup comparisons on neurodevelopmental assessment scores. *Post-hoc* unpaired *t*-tests were carried out to identify significant differences between groups. Significance level was set to α = 0.05. Alpha adjustment for multiple comparisons was done according to false discovery rate (47).

## Results

### Patients' Characteristics

A total of 80 participants were included in this study: 41 children in the *Surveillance Group* and 39 in the *Historical Control Group*. Perinatal, surgical, critical, and demographic characteristics of the participants are presented in Table 1. The most common heart defects were transposition of the great arteries (39% in the *Surveillance Group* and 31% in the *Historical Control Group*), coarctation of the aorta (14% in the *Surveillance Group* and 28% in the *Historical Control Group*), and Tetralogy of Fallot (17% in the *Surveillance Group* and 18% in the *Historical Control Group*). We found a statistical tendency for a greater proportion of children of the *Surveillance Group* who required cardiopulmonary bypass during surgery compared to the *Historical Control Group* (*P* = 0.084). There were no other differences in perinatal, surgical, critical, and demographic characteristics between groups.

**Table 1:** Clinical and demographical characteristics of infants with CHD.

		**Groups**		
	**Total (*N* = 80)**	**Surveillance (*n* = 41)**	**Control (*n* = 39)**	***p*-values**
Male sex, *n*	37 (46.3)	17 (41.5)	20 (51.3)	0.38
Prenatal diagnosis, *n*	41 (60.3)	23 (69.7)	18 (51.4)	0.12
Gestational age at birth, *weeks*	38.7 (1.8)	38.5 (1.8)	38.9 (1.9)	0.31
Birth weight, *kg*	3.2 (0.6)	3.08 (0.6)	3.2 (0.7)	0.23
Apgar score at 5 min	8.5 (1)	8.5 (1.1)	8.6 (0.9)	0.63
Confirmed genetic abnormality, *n*	7 (9.3)	5 (13.5)	2 (5.3)	0.22
Cyanotic heart lesions, *n*	46 (57.5)	26 (63.4)	20 (51.3)	0.27
Anatomic classification of CHD^a^, *n*				0.35
Class I	50 (62.5)	28 (68.3)	22 (56.4)	
Class II	25 (31.3)	10 (24.4)	15 (38.5)	
Class III	2 (2.5)	1 (2.4)	1 (2.6)	
Class IV	3 (3.8)	2 (4.9)	1 (2.6)	
Primary cardiac surgery				
Age at surgery, days	18 (67)	27 (143)	13 (30.8)	0.09
Cardiopulmonary bypass, *n*	54 (76.1)	32 (84.2)	22 (66.7)	0.08
Cardiopulmonary bypass time, *min*	165 (80.4)	177 (100)	147.5 (32.2)	0.19
Open chest after surgery, *n*	20 (31.3)	13 (37.1)	7 (24.1)	0.26
RACHS-1 score^b^^,^ ^c^, *n*				0.29
Category 1	3 (3.8)	0 (0.0)	3 (8.1)	
Category 2	30 (38.5)	16 (39.0)	14 (37.8)	
Category 3	25 (32.1)	17 (41.5)	8 (21.6)	
Category 4	18 (23.1)	6 (14.6)	12 (32.4)	
Category 6	2 (2.6)	2 (4.9)	0 (0.0)	
Primary hospital admission				
Hospital length stay, *days*	17 (23)	20 (23)	16 (21.8)	0.44
Pediatric intensive care unit length stay, *days*	6.5 (5)	7 (5.5)	6 (5.3)	0.78
Maternal education level, *n*				0.75
High school	24 (30.4)	13 (31.7)	11 (28.9)	
Vocational school	21 (26.6)	11 (26.8)	10 (26.3)	
College/university	34 (43)	17 (44.7)	17 (41.5)	
Age at neurodevelopmental assessment, *months*	44.1 (1.2)	44.3 (1.2)	43.9 (1.2)	0.13

*Data expressed as number of participants (percentages) for dichotomous and categorical variables and mean (SD) for continuous variables. Data for age at surgery, hospital length stay, and PICU length stay are expressed as median (IQR)*.

a *Anatomic classification of CHD defined according to Clancy et al. (45). Class I: two-ventricle heart without arch obstruction; class II: two-ventricle heart with arch obstruction; class III: single-ventricle heart without arch obstruction, and class IV: single-ventricle heart with arch obstruction (45)*.

b *Data unavailable for two participants of the Historical Control Group*.

c *Surgical risk category defined according to Jenkins and Gauvreau (46)*.

We found no differences between the 80 participants (41 in the *Surveillance Group* and 39 in the *Historical Control Group*) and children who declined participation in the study (16 CINC patients and 23 children born before the CINC was established; all *P* > 0.05) regarding sex or medical characteristics (i.e., gestational age at birth, prenatal diagnosis, anatomic CHD classification, birth weight, APGAR score at 5, age at surgery, CPB time, hospital length stay, PICU length stay, and open chest after surgery). No data were available regarding socio-economic status for those who declined participation.

Based on parental reports, 14 (37%) children in the *Historical Control Group* received at least one remedial service before 3 years of age through references within standard health care. As expected, this rate was smaller compared to the *Surveillance Group*, in which 37 (90%) children received at least one remedial service before the age of 3, χ^2^ = 24.5, *P* < 0.001. Among the 14 children of the *Historical Control Group* who received services, only 7 (50%) had met more than one type of health professional, whereas 31 (84%) children in the *Surveillance Group* had been followed by at least two different health professionals, χ^2^ = 6.1, *P* = 0.013. Physical therapists, occupational therapists, speech-language pathologists, and nutritionists were the most consulted professionals in both groups. Among the 20 (49%) children in the *Surveillance Group* who received speech and language therapy, 30% received only indirect intervention, such as recommendations of daily activities to stimulate language development, whereas 70% received both indirect and direct speech therapy. In comparison, only 5 (13%) children in the *Historical Control Group* received services from a speech-language pathologist. A detailed description of remedial services received in both groups is presented in Table 2.

**Table 2:** Participants' characteristics relating to the use of remedial services.

	**Groups**	
	**Surveillance (*n* = 41)**	**Control (*n* = 38)^a^**
Ever received remedial services, *n*	37 (90)	14 (36.8)
Number of remedial services, *n*		
One	6/37 (16.2)	7/14 (50)
Two	16/37 (43.2)	3/14 (21.4)
Three	8/37 (21.6)	3/14 (21.4)
Four	6/37 (16.2)	1/14 (7.1)
Five	1/37 (2.7)	0/14 (0)
Type of remedial services, *n*		
Speech-language therapy	20/41 (49)	5/38 (13)
Physical therapy	37/41 (90)	7/38 (18)
Occupational therapy	15/41 (37)	8/38 (21)
Psychology therapy	0/41 (0)	2/38 (5)
Special educational support	3/41 (7)	1/38 (3)
Nutrition	17/41 (42)	4/38 (11)

*Data expressed as sample size (percentages)*.

a *Data unavailable for one participant of the Historical Control Group*.

### Cognitive, Motor, and Behavioral Results

The mean age at testing was similar in both groups (*P* = 0.129) with assessments at 43.9 months (SD = 1.2) for the *Historical Control Group* and at 44.3 months (SD = 1.2) for the *Surveillance Group*. Table 3 shows groups' mean scores for cognitive, motor, and behavioral measures. Each mean score, including indices and subtests, was in the normal range, except for the Zoo Locations subtest and the Working Memory Index, which were both in the high average for the *Historical Control Group*. Although in the normal range, distributions of gross motor scores were slightly down shifted with an increased proportion of children performing under clinical cutoffs for both groups [12 (31%) from the *Historical Control Group* and 7 (19%) from the *Surveillance Group* performed equally to or below the 16th percentile (1 SD or more below norms)].

**Table 3:** Neurodevelopmental outcome of children with CHD.

	**Groups**				***p*-values**
	**Surveillance (*****n*** **=** **41)**		**Control (*****n*** **=** **39)**	
	**Mean of scores (SD)**	**Missing data *n* (%)**	**Mean of scores (SD)**	**Missing data *n* (%)**	
**Cognition (WPPSI-IV)**					
Full-scale IQ (FSIQ)	100 (11.7)	4 (9.8)	100.9 (13)	0 (0)	ns
Verbal comprehension index	93.3 (12.1)	1 (2.4)	97.4 (12.1)	0 (0)	ns
Receptive vocabulary	11.4 (3.5)		9.5 (2.4)		0.048
Information	8.8 (3.2)		10 (2.9)		ns
Picture naming	9 (3)		10 (2.4)		ns
Visual spatial index	103.8 (13.5)	3 (7.3)	100.54 (11.6)	0 (0)	ns
Block design	10.1 (2.5)		10.4 (2.2)		ns
Object assembly	11.3 (2.8)		10 (2.8)		0.080
Working memory index	102.45 (12. 6)	8 (19.5)	111.1 (13.4)	7 (17.9)	0.032
Picture memory	10 (2.5)		10.9 (2.9)		ns
Zoo locations	10.8 (3.2)		12.3 (2.9)		0.080
**Gross motor (PDMS-2)**					
Gross motor quotient	94.6 (9.2)	5 (12.2)	94.1 (11.2)	0 (0)	ns
Stationary	9.4 (2.1)		9.1 (2.4)		ns
Locomotion	9.2 (2)		9.5 (2.3)		ns
Object manipulation	8.9 (1.6)		8.6 (1.5)		ns
**Fine motor (M-ABC-2)**					
Manuel dexterity score	97.7 (12.3)	2 (4.9)	99.1 (14)	0 (0)	ns
Posting coins	9.6 (2.39)		9.4 (3.2)		ns
Threading beads	9.5 (3.15)		9.4 (2.9)		ns
Drawing trail	8.8 (3)		9.6 (3.4)		ns
**Behavior (BASC-2)**		10 (24.4)		6 (15.4)	
Externalizing problems	48.5 (9)		51.1 (8.4)		ns
Internalizing problems	55.3 (10.5)		53.4 (8.9)		ns
Behavioral symptoms index	48.3 (8.65)		48.5 (7)		ns
Adaptative skills	54 (6.55)		54.9 (7.4)		ns

*Data expressed as mean (SD). Mean performances of 10 (SD = 3) and 100 (SD = 15) are expected on subtest and scale levels, respectively. p-values were calculated using unpaired sample t-tests with alpha adjustment according to false discovery rate*.

*WPPSI-IV, Wechsler Preschool and Primary Scale of Intelligence, Fourth Edition; PDMS-2, Peabody Developmental Motor Scale, Second Edition; M-ABC-2, Movement ABC, Second Edition; BASC-2, Behavior Assessment System for Children, Second Edition*.

### Differences Between Groups

The one-way MANOVA revealed significant differences between groups for performances on cognitive subtests, with a clinically significant effect size, *F*_(6, 57)_ = 5.491, *P* < 0.001; Wilk's Λ = 0.634, partial η^2^ = 0.366. *Post-hoc t*-tests revealed that the *Surveillance Group* performed better than the *Historical Control Group* (*P* = 0.048) on the Receptive Vocabulary subtest. Individual results revealed that 3 (7%) children from the *Surveillance Group* performed equally to or below the 16th percentile (1 SD below mean), compared to 6 (16%) from the *Historical Control Group* on this subtest. *t*-tests also showed a statistical trend toward a greater score on the Object Assembly subtest and a lower score on the Zoo Locations subtest in the *Surveillance Group* compared to the *Historical Control Group* (both *P* = 0.08). We also found a significant group difference in cognitive primary indices, *F*_(4, 49)_ = 3.449, *P* = 0.013; Wilk's Λ = 0.810, partial η^2^ = 0.190. *Post-hoc* comparisons indicated that the mean score for the Working Memory Index was significantly higher for the *Historical Control Group* compared to the *Surveillance Group* (*P* = 0.032), which is coherent with the results obtained on the subtest level. We found no significant effect of group on gross and fine motor scores as well as behavioral scales.

## Discussion

Following the American Heart Association and the American Academy of Pediatrics recommendations of systematic developmental surveillance, screening, and evaluation of children with CHD (30), many neurocardiac developmental follow-up programs have emerged worldwide. The feasibility of implementing such programs has been demonstrated (26, 48) and factors improving follow-ups have been investigated (49, 50). The benefits of early intervention in supporting neurodevelopment have been shown in other clinical pediatric populations (e.g., preterms, autistic children) (51–55). Despite variability with regard to the type of interventions (provided at a clinic vs. home-based interventions) or professionals involved, studies have demonstrated a positive impact of early intervention programs compared to standard health care in enhancing cognitive and behavioral outcomes of children born preterm (55–58). In comparison, literature on interventional effect in CHD is only emerging but is gathering increasing attention. The aim of the present study was to assess the impact of the CINC early systematic interdisciplinary developmental follow-up program on neurodevelopmental outcomes of preschoolers with critical CHD compared to standard health care.

### Cognitive and Language Skills

Children of the *Surveillance Group* and the *Historical Control Group* demonstrated similar global intellectual functioning. We found a significant difference, with clinically significant effect size, in performances on the receptive vocabulary subtest and a statistical tendency on the visuo-constructive subtest between groups. These skills have been previously reported to be impaired in children with CHD (16, 19, 59–61). These results suggest an advantage of the children who took part in the CINC early systematic interdisciplinary developmental follow-up program regarding receptive language competency compared to children of the *Historical Control Group* who received standard health care.

As part of their developmental follow-up and based on the MBCDI screening results, 20 (49%) children of the *Surveillance Group* were referred to a speech-language pathologist for an extensive language assessment before the age of 3, with 70% who received both direct speech therapy and indirect intervention in the form of recommendations to stimulate language development. In comparison, only 5 (13%) parents of children from the *Historical Control Group* reported receiving services from a speech-language pathologist. These children were referred only when a language delay was already observed or if parental concerns were present, whereas all children of the *Surveillance Group* underwent early systematic language screening, which may result in earlier detection of language impairments and higher referral rates. The individualized intervention received by children from the *Surveillance Group* may have strengthened the development of their receptive language skills, as illustrated by a lower proportion of receptive difficulties at the age of 3 (7% in the *Surveillance Group* compared to 16% in the *Historical Control Group* being below 1 SD).

Additionally, we found that the children from the *Surveillance Group* tend to have better visuo-constructive skills compared to children from the *Historical Control Group*. Occupational therapists of the CINC sometimes recommended visuospatial and visuo-constructive activities for parents to perform with their child, including shape sorting games and stacking toys, to help stimulate perceptual–motor development and hand–eye coordination abilities. However, it is not possible at this point to state that we found an effect of the developmental follow-up program or the specific individualized intervention on these skills. This potential benefit in preserving the development of visuo-constructive skills remains to be demonstrated. Nonetheless, these results allow us to flag this set of competencies for further assessment at a growing age.

Beyond the benefits of direct intervention, literature has previously demonstrated the effects of a more global approach in improving neurocognitive functions in children at risk for developmental delay. For instance, several studies have demonstrated that intervention programs including parental support could benefit the neurodevelopment of preterm children (55, 58, 62, 63). In the CHD population, McCusker et al. (64) have documented better cognitive and communicative abilities in 8-month-old infants with CHD whose mother participated in the Congenital Heart Disease Program. Through psychoeducation, coaching, and therapy, this psychosocial program notably aimed at promoting child development by supporting maternal adjustment to the diagnosis and teaching effective coping strategies. The Congenital Heart Disease Program also demonstrated gains on measures of maternal mental health and family functioning, which may indirectly influence the child outcomes (65). In the CINC program, psychoeducational support is systematically offered to parents. Every visit is also an opportunity for parents to ask questions to pediatric specialists. If needed, the nurse practitioner coordinator also addresses parental concerns and offers additional support. In addition to the potential impact of the individualized direct intervention, the affective and psychoeducational support given to parents may have had a beneficial effect on the neurodevelopment of children with critical CHD. Additional research is needed to investigate the potential benefits of the CINC program in improving family well-being.

We found that children of the *Surveillance Group* showed a significantly lower mean score on the Working Memory Index compared to the children of the *Historical Control Group*. However, this result should be taken with caution as we cannot exclude that the high rate of missing data on this scale could have biased group comparisons (see Table 3). For a substantial number of children, we were not able to obtain adequate collaboration to acquire valid data for the two subtests composing this index (Zoo Locations and Picture Memory). As frequently observed by assessors in both groups, these two subtests specifically appeared to require substantial efforts from the participants. The high number of missing values could have resulted in an over-representation of the highest performances, potentially explaining the mean performance in the high average at the Working Memory Index for the *Historical Control Group*. This could also have reduced statistical power, thus not reflecting a true difference on memory span and working memory skills between groups.

### Gross and Fine Motor Functions

Overall, gross motor assessment revealed mean scores in the normal range. However, this developmental domain still appears as a specific area of concern, with 31% of the *Historical Control Group* and 19% of the *Surveillance Group* performing equally to or below the 16th percentile (1 SD or more below norms). In both groups, gross motor disabilities were characterized by difficulties with standing balance, decreased lower limbs strength, proximal instability, and difficulty with hand–eye coordination. Although some studies document a gradual improvement in gross motor abilities up to the age of 3, difficulties in this domain persist at school entry where gross motor impairment rate generally exceeds normative expectations (9, 66).

We found no significant differences between groups for gross motor functions. Since gross motor impairments are known to be the first manifestations of altered neurodevelopment in infants with CHD, it is possible that family physicians and pediatricians, even outside specialized clinics, may be successful at detecting these difficulties and thus accurately referred patients to relevant rehabilitation services or offer parental recommendations on how to foster the child's motor development (67). Based on parental reports, 18% of children in the *Historical Control Group* had received treatments with a physical therapist in addition to the standard health care, physical therapy being one of the most frequently received interventional services. This could have preserved their gross motor skills and may explain the absence of significant differences between groups. As discussed by Kynø et al. (68) in the preterm population, the standard care offered to all patients with CHD at the pediatric intensive care unit (e.g., nesting, post-surgical positioning program, parents present as much as possible, etc.) may also have supported the child's development, therefore reducing outcome differences between groups. Nevertheless, the greater proportion of children in the *Historical Control Group* performing below the 1 SD cutoff suggests that children who did not undergo early systematic interdisciplinary developmental follow-ups are at higher risk for gross motor impairments compared to children who took part in the CINC program. We cannot exclude either that this absence of significant statistical differences in gross motor skills could be due to the small sample size.

Regarding fine motor functions, occupational therapists have observed limitations in the proximal stability of the upper limbs in a significant proportion of children from both groups. These limitations caused distal tremors and difficulties with motor coordination. Children seemed to succeed in compensating for these difficulties with both groups performing within the normal range in fine motor tasks. However, children frequently exhibited non-voluntary mouth movements, revealing that these tasks required substantial efforts. In future studies, systematic video recordings of children's evaluations could help analyze qualitative observations from specialists and thus allow the tracing of a more subtle neurodevelopmental profile. We hypothesize that these subtle limitations may progress into significant deficits with growing environmental demands. Conducting a follow-up at school age is thus crucial to document the developmental trajectory and to detect motor difficulties that could arise and significantly impact schooling and social life (9).

### Behavioral Functioning

Children of the *Surveillance Group* and the *Historical Control Group* demonstrated similar results on the BASC-2, revealing behavioral functioning in the normative range for both groups. At that age, only 2 children (5%) of the *Historical Control Group* and no children from the *Surveillance Group* received services from a psychologist. However, as it is integrated within the CINC systematic program, psychologists conduct the 24-month-old developmental evaluation and thus have the possibility to provide parental counseling and psychological support without the need to refer the family to external psychological therapy. During the evaluation, assessors observed behavioral regulation issues (e.g., oppositional behaviors, hyperactivity, and difficulties to mobilize cognitive resources) that have not been reported in the parental questionnaire. Based on our experience and the literature, behavioral issues appear gradually with age in children with CHD and become more salient to parents when the child enters school. We are following these children and expect parents to report more behavioral issues at school age.

### Use of Remedial Services

Referral rates for interventional services were higher in children who took part in our interdisciplinary developmental follow-up program (90%) compared to children who received standard health care (37%). These referral rates are in accordance with a previous study we conducted on a subsample of CINC patients where we reported that 79% were identified at the age of 4 months to be at high risk for gross motor delay and were referred to the CINC physical therapist for interventional treatment (35). In the current literature, apart from recognizing that the prevalence of children with CHD who need interventional services largely exceeds that of healthy children, there is no clear consensus regarding the referral rate for additional care, which depends on available public health resources as well as insurance coverage (50). For instance, Mussatto et al. (39) reported that 74% of 3-year-old children with CHD received remedial services from US regional early intervention programs or private therapy, and Calderon et al. (69) indicated that 53% of 5-year-old children with CHD from the Paris area received at least one rehabilitation service. Another study revealed that 40–95% of 8-year-old children with CHD who exhibited specific developmental delays did not receive the relevant services (28). In light of these findings, we think that an early systematic developmental follow-up program results in a potential earlier detection of neurodevelopmental impairments associated with CHD, thus generating higher referral rates and at an earlier age. However, this remains to be documented, and the relevance of higher referral rates to be demonstrated in future studies.

### Clinical Characteristics

While no statistical differences were found for most perinatal, surgical, and critical characteristics between groups, a greater proportion of children of the *Surveillance Group* required cardiopulmonary bypass during surgery. This factor has been shown to be associated with worse neurodevelopmental outcomes (61, 70, 71). Children from the *Surveillance Group* may have been at higher risk of neurologic sequelae after surgery, thus affecting their neurodevelopmental outcome. The higher neurological burden of the CINC patients may have contributed to blurring group differences. More clinically equivalent groups may lead to greater effects of interventional treatment between groups.

### Limitations

First, the small sample size may have prevented us from clearly demonstrating an advantage of the interdisciplinary developmental follow-up program over the existing standard health care, and limits the generalization of our findings to the whole complex CHD population. The sample size was restricted in accordance with the hospital's flow of patients with CHD. Specifically, the *Historical Control Group* was recruited and tested at a time period overlapping the beginning of the CINC program. Therefore, we reached a maximal sample size since all children subsequently born with a critical CHD were referred to the CINC. Second, the use of a historical control group may have introduced biases due to a different time of enrollment. Although no major changes in cardiac care (e.g., cardiac surgeon) occurred in the hospital over this timeframe, subtle changes may have influence group differences. We might also have conducted the evaluation too early in the developmental stage to observe significant differences in high-level cognitive functions, fine motor abilities, and behavior. We cannot exclude that the CINC program may have longer-term impacts that would be more precisely measured in an older cohort as opposed to preschoolers, thus stressing the importance of following these children up to school age and even later on. Finally, because evaluations were performed by the CINC professionals and served for the clinical follow-up of the CINC patients, it is to be noted that examiners were not blind to the children's group, which might have induced an assessment bias.

### Conclusions

Results suggest that the CINC early systematic and interdisciplinary developmental follow-up program might prevent the emergence of receptive language difficulties in preschoolers. While these results are promising, they remain subtle at this age and several factors limit their generalization. It is therefore crucial to assess the impact of early systematic developmental follow-ups at school age when this impact might be greater and higher-order functions and learning abilities can be assessed. At the CINC, subsequent follow-ups occur at 5 and 8 years of age, with extensive motor, cognitive, and language assessments performed by occupational therapists, neuropsychologists, and speech-language pathologists, respectively. In addition, we are currently following the children from the *Historical Control Group*, who are now starting to turn 8 years, and we have been able to recruit more children in this group to increase statistical power. This second phase of the current study will allow us to more accurately assess the impact of our early systematic developmental program at school age.

While neurodevelopmental assessments show that cognitive, motor, and behavioral development of children with critical CHD appears globally within the normal range prior to entry at school, it is not to exclude that subtle limitations may progress into deficits at school age with growing environmental demands. Early systematic assessments with an interdisciplinary team therefore appear to be of great importance to document the developmental trajectory of these functions and to detect subtle impairments that may have a functional impact on behavior, learning, social functioning, and quality of life.

## Data Availability Statement

The datasets generated for this study will not be made publicly available due to patient confidentiality.

## Ethics Statement

The studies involving human participants were reviewed and approved by the Institutional Research Ethics Board of the Sainte-Justine University Hospital Center. Written informed consent to participate in this study was provided by the participants' legal guardian/next of kin.

## Author Contributions

SF participated in designing the study and data collection, conducted the analysis, drafted the initial manuscript, and reviewed and revised the manuscript. LC-D participated in data collection and analysis, drafted the initial manuscript, and reviewed and revised the manuscript. M-NS contributed to the analysis and reviewed and revised the manuscript. AD, KG, LD, ÉP, and JP contributed to the design of the study, collected the data, and reviewed and revised the manuscript. SP, AB-T, and IC contributed to the data collection and reviewed and revised the manuscript. NP contributed to the design of the study and reviewed and revised the manuscript. AG conceptualized and designed the study, supervised the collection of the data, contributed to the draft of the initial manuscript, and revised and critically reviewed the manuscript for important intellectual content. All authors approved the final manuscript as submitted and agree to be accountable for all aspects of the work.

## Conflict of Interest

The authors declare that the research was conducted in the absence of any commercial or financial relationships that could be construed as a potential conflict of interest.
